# Patterns of small involuntary fixation saccades (SIFSs) in different neurodegenerative diseases: the role of noise

**DOI:** 10.1007/s00221-023-06633-6

**Published:** 2023-05-29

**Authors:** Wolfgang Becker, Anna Behler, Olga Vintonyak, Jan Kassubek

**Affiliations:** 1 Section of Neurophysiology, Department of Neurology, University of Ulm, Ulm, Germany; 2 Department of Neurology, University of Ulm, Ulm, Germany

**Keywords:** Saccadic intrusions, Small involuntary fixation saccades, Square wave jerks, SWJ coupling, Amyotrophic lateral sclerosis, Progressive supranuclear palsy, Physiological and technical noise

## Abstract

During the attempt to steadily fixate at a single spot, sequences of small involuntary fixation saccades (SIFSs, known also as microsaccades οr intrusions) occur which form spatio-temporal patterns such as square wave jerks (SWJs), a pattern characterised by alternating centrifugal and centripetal movements of similar magnitude. In many neurodegenerative disorders, SIFSs exhibit elevated amplitudes and frequencies. Elevated SIFS amplitudes have been shown to favour the occurrence of SWJs (“SWJ coupling”). We analysed SIFSs in different subject groups comprising both healthy controls (CTR) and patients with amyotrophic lateral sclerosis (ALS) and progressive supranuclear palsy (PSP), i.e. two neurodegenerative diseases with completely different neuropathological basis and different clinical phenotypes. We show that, across these groups, the relations between SIFS amplitude and the relative frequency of SWJ-like patterns and other SIFS characteristics follow a common law. As an explanation, we propose that physiological and technical noise comprises a small, amplitude-independent component that has little effect on large SIFSs, but causes considerable deviations from the intended amplitude and direction of small ones. Therefore, in contrast to large SIFSs, successive small SIFSs have a lower chance to meet the SWJ similarity criteria. In principle, every measurement of SIFSs is affected by an amplitude-independent noise background. Therefore, the dependence of SWJ coupling on SIFS amplitude will probably be encountered in almost any group of subjects. In addition, we find a positive correlation between SIFS amplitude and frequency in ALS, but none in PSP, suggesting that the elevated amplitudes might arise at different sites in the two disorders.

## Introduction

The attempt to quietly fixate at a visual object is repeatedly interrupted by a variety of eye movements comprising slow drifts (typically ≤ 0.07°/s) and small flicks ranging in amplitude from 0.01° to about 2°. Early studies of fixation using suction lenses with mirrors for registration focused on flicks of less than about 0.25° (e.g. Ditchburn and Ginsborg 1953) which came to be known as microsaccades. With the advent of other low noise recording methods (e.g. video-oculography), larger flicks with amplitudes up to about 2°, referred to as saccadic intrusions (Leigh and Zee 2006), became the subject of many studies. The distinction between microsaccades and saccadic intrusions is an artificial one, based solely on arbitrary amplitude ranges. In fact, microsaccades, saccadic intrusions, and voluntary refixation saccades of up to 60° form a continuum by many criteria (Hafed 2011; Martinez-Conde and Macknik 2015). Therefore, we here subsume microsaccades and saccadic intrusions under the common term 'small involuntary fixation saccades*'* (SIFSs).

**Fig. 1:**
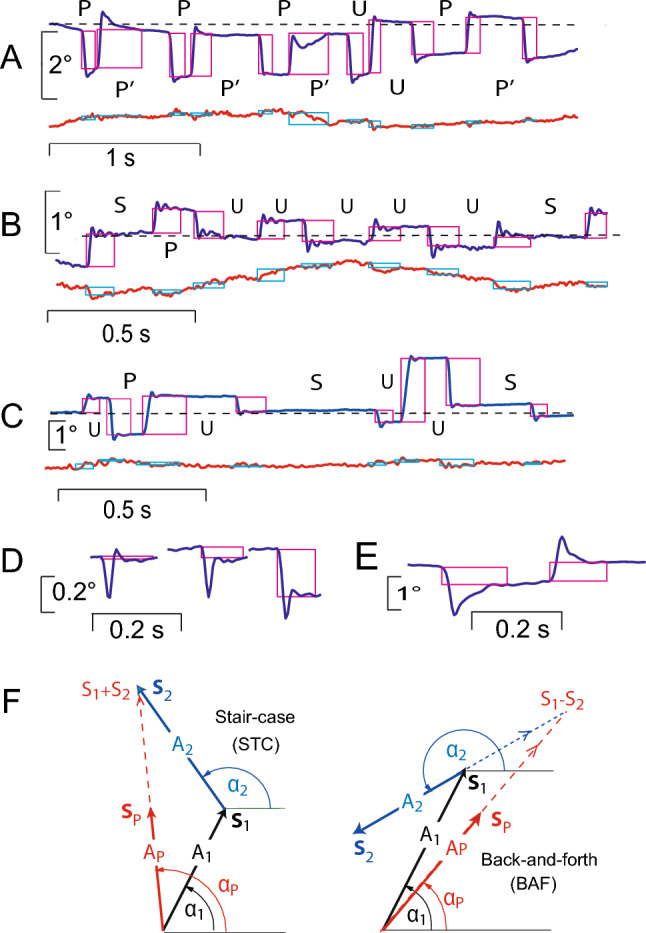
Method of analysis of SIFSs **A**–**E** and vector representation of SIFS patterns **F**. Blue traces, horizontal eye position; red, vertical. Magenta and light blue rectangles reflect results of interactive analysis; left edges mark onset times of SIFSs, right edges capture start of the postsaccadic steady-state position; heights indicate net displacement. Dashed horizontal lines mark approximate fixation point position. Pattern types formed by successive SIFSs are indicated by letters between the constituent SIFSs; S, stair case; U, unpaired back-and-forth; P, paired back-and-forth pattern forming 'classical' SWJs; P', paired back-and-forth patterns that do not constitute SWJs. **A** PSP patient with large amplitudes and correspondingly high PBF frequency. **B** ALS patient with small SIFS amplitudes and, hence, low PBF frequency (postsaccadic oscillation is neurally generated and not an artefact). **C** Control subject; note grossly overshooting 'corrections' after small centrifugal SIFSs resulting in bursts of three SIFSs. **D** PSP patient; left SIFS out of series of three consecutive SIFSs (intervals cut out) demonstrates the possibility that SIFSs have virtually zero amplitude by our definition. **E** PSP patient; amplitude determination in cases of SIFS variants with dynamic overshoot, followed by a slow decay to the final steady-state position. **F** Vector representation of individual SIFSs and of patterns. Vectors **S**_i_ with amplitudes and orientations (A_*i*_, α_*i*_) represent leading (*i* = 1) and trailing SIFS (*i* = 2) of pattern, respectively. Pattern vectors **S**_P_ with amplitude and orientation (A_P_, α_P_) equal vectorial average of **S**_1_ and **S**_2_ (staircase pattern) or of **S**_1_ and -**S**_2_ (back and forth), respectively

In healthy humans, the SIFSs occurring during fixation at a small target spot on a uniform background mostly have an approximately horizontal orientation, with an average amplitude of about 0.5° and a frequency of occurrence of about 1 s^−1^. Typically, the spatio-temporal pattern formed by consecutive SIFSs (e.g. **S**_n_, **S**_n-1_, **S**_n+2_, …) consists of an initial centrifugal **S**_n_ away from the fixation target followed, after a brief period of 200-400 ms, by a centripetal **S**_n+1_ towards the target. This sequence is repeated after variable delays throughout the fixation attempt. So-called square wave jerks (SWJs) result if the centrifugal and centripetal SIFSs have 'similar' magnitudes and motion planes (Fig. 1A). In some subjects, bursts of three SIFSs may repetitively occur where **S**_n+1_ crosses the required line of sight and **S**_n+2_ attempts to returns to it (Fig. 1C). Finally, **S**_n_ and **S**_n+1_ may also have the same direction, thus forming a staircase pattern.

The function of *centripetal* SIFSs seems to be obvious, that is, to correct the fixation errors created by the preceding *centrifugal* SIFSs or by slow eye drifts (Nachmias 1959). However, there is still no unanimously agreed answer as to why centrifugal SIFSs arise at all and make these corrections necessary. It has been suggested that they might be important for visual information processing or the prevention of image fading, whereas other authors point out that these functions may be merely dispensable by-products of SIFSs or that SIFSs may even disturb vision (for reviews of these controversies see Collewijn and Kowler 2008; Hafed 2011; Poletti and Rucci 2010; Rolfs 2009). More recently, SIFSs have been shown to reflect shifts of covert attention (Engbert and Kliegl 2003; Hafed and Clark 2002) and to be paralleled by corresponding shifts of activity on the collicular motor map (Hafed et al. 2009; Hafed 2011), thus offering a powerful tool for relating neuropsychological phenomena to neural processes. Yet, these findings still do not answer the question whether SIFSs serve an indispensable function or merely constitute an occasionally useful epiphenomenon. It is because of this open question that we chose the neutral term 'SIFS' which avoids the connotation of disturbance conveyed by the term 'intrusion' and respects the classical definition of 'microsaccade' as referring to flicks of less than 0.25°.

As far as examined, all neurodegenerative diseases affecting the cortical, subcortical and cerebellar structures appear to cause larger and/or more frequent SIFSs as compared to healthy subjects. Among others, this holds for Parkinsonian syndromes (Otero-Millan et al. 2011; Pinnock et al. 2010) and amyotrophic lateral sclerosis (Donaghy et al. 2009; Shaunak et al. 1995). Large SIFS amplitudes are known to favour the occurrence of SWJ-like SIFSs ('SWJ coupling') in healthy controls and Parkinsonian disorders including progressive supranuclear palsy (PSP), and also in recessive cerebellar ataxia (Otero-Millan et al. 2013), a disease unrelated to Parkinsonism. We here ask whether this dependence of SWJ coupling on SIFS amplitude occurs also in other non-parkinsonian neurodegenerative diseases and, if so, whether it can be described by a unifying law that would hold across both healthy subjects and various neurodegenerative conditions. The demonstration of such a common law might lead to an explanation of why large SIFS amplitudes favour the occurrence of SWJ coupling. To pursue this issue, we consider the SIFS patterns occurring in CTR and in the neurodegenerative diseases ALS and PSP. Because the amplitude range of ALS overlaps at one end with that of CTR and at the other end with that of PSP, one can examine whether, given similar SIFS amplitudes, the three groups of subjects exhibit similar relative frequencies of SWJ-like patterns. Published relationships between SWJ likelihood and SIFS amplitude (Otero-Millan et al. 2013) do not seem to support such a conjecture but are based on a small sample of subjects only.

From a clinical point of view, the two diseases that are being compared here are completely different. ALS is primarily a motor neuron disease causing muscle weakness and ultimately a complete paralysis; because it entails also cognitive and behavioural changes which appear early during its course (Crockford et al. 2018; Lulé et al. 2018), it is regarded as a multisystem disorder. The disease is caused by aggregations of the DNA-binding protein pTDP-43 which propagate in a four-stage process from the motor neocortex, affecting sequentially the spinal cord and brainstem, then the frontal and parietal lobes, and ultimately the anteromedial temporal lobes. (Braak et al. 2013; Brettschneider et al. 2013). PSP was previously considered to be characterised by the eponymous supranuclear palsy of vertical eye movements, postural instability with falls early in the disease course, axial rigidity, apathy, and executive dysfunction (Steele et al. 1964). PSP is now recognised as a range of movement, behavioural, and language syndromes which may not necessarily include eye movement deficits in some cases. PSP is pathologically characterised by the deposition of a characteristic four-repeat tau protein leading to neuronal loss and gliosis in the brainstem and in subcortical and cortical structures including the frontal lobes (Kovacs et al. 2020). Different clinical subtypes have been described beyond the ‘classical’ presentation as Richardson’s syndrome (PSP-RS), including the subtype PSP-parkinsonism (PSP-P), and also various other clinical phenotypes (Höglinger et al. 2017; Respondek et al. 2017). Although ALS and PSP are fundamentally different diseases, their impact on the frontal cortex constitutes a common trait which manifests itself by the various cognitive and behavioural disorders mentioned above.

## Methods

### Participants

The present report is a retrospective study based on data from 48 control subjects (CTR) without neurological, psychiatric or other medical conditions, 94 ALS patients, and 50 PSP patients (all groups matched for age; Table 1). The diagnosis of ALS was obtained according to the revised El Escorial criteria (Ludolph et al. 2015). The diagnosis of recent PSP cases was based on the MDS-PSP criteria (Höglinger et al. 2017); these were also retrospectively applied to cases diagnosed originally according to the NINDS-SPSP criteria (Litvan et al. 1996). The SIFSs data of both patient groups stem from a fixation paradigm that was run as part of a standardised oculomotor test battery administered during the patients' clinical workup. Control subjects without known neurological impairments were recruited among patients’ relatives and acquaintances of the authors and were presented with the same test battery as the patients. In part, the control and ALS cohorts are identical to those of a preliminary study of SIFSs in ALS (Becker et al. 2019); some younger subjects were replaced by older ones to better match the age of the PSP cohort. The study was in accordance with the Declaration of Helsinki, had been approved by the ethics committee of the University of Ulm, and subjects had given their written informed consent.

**Table 1:** Demographic data

Group	*N*	m/f	Age [years] Median (Q05–Q95)	Number of patients in subtype groups	Duration of disease [months] Mean (min–max)	ALS: ALSFSR-R PSP: UPDRS Mean (min–max)
CTR	47	1.18	66.6 (51–77)	–	–	–
ALS	94	1.14	63.8 (51–78)	Sp 71; Bu 23	15 (2–59)	40.1 (18–48)
PSP	50	0.85	70.0 (53–79)	PSP-R, 42; PSP-P, 8	36 (6–90)^a^	30.1 (15–50)

*N* number of subjects, *Q05* (Q95), *5%* *(95%)* quantiles, *m/f* male to female ratio, *ALSFSR_R* revised ALS functional rating scale, *UPDRS* unified Parkinson’s disease rating scale, *Sp* spinal onset ALS, *Bu* bulbar onset ALS, *PSP-R* Richardson type, *PSP-P* Parkinson type

^a^Data based on 32 patients

### Equipment and procedures

Participants were seated in a comfortable chair at the centre of a white hemicylindrical screen, which carried arrays of red light-emitting diodes (LEDs; invisible when not lit) subtending 0.3° of visual angle (eye-to-screen distance of 1.50 m). They rested their heads on a chin support and were donned with a head-mounted video-oculography system (VOG; EyeSeeCam®, nominal horizontal resolution 0.05°–0.1°), which sampled the movements of both eyes at a frequency of 220 Hz, displayed them online for monitoring, and transferred them to Matlab® files. After adjusting the VOG cameras, the room lighting was dimmed to a standardised low-illumination level, and participants were presented with sinusoidal movements of a light spot (projected via mirror galvanometers) in the horizontal plane (frequency 0.125 Hz, amplitude ± 20°), followed by vertical movements (± 15°) to elicit slow smooth pursuit movements serving calibration. The central LED then was lit for 32.1 s and participants were asked to steadily fixate at it while their SIFSs were recorded. Thereafter, reactive saccades to horizontal and vertical target steps of various magnitude were elicited; the responses to 5 or 10° steps were used for calibration when patients were unable to follow the full sinusoidal excursions.

Data analysis was carried out with dedicated in-house Matlab® programmes and Excel® macros. The calibrated recordings of horizontal and vertical eye position from both eyes were converted into cyclopean signals by an averaging procedure and displayed on a computer screen. SIFSs were identified by visual inspection based on their characteristic dynamic overshoots. Using the larger of the two eye movement components (mostly the horizontal one), the evaluator positioned a vertical hair line at saccade onset and a second one at the end of the dynamic overshoot processes thus capturing the final steady state displacement (cf. Figure 1A–E). The horizontal and vertical eye positions at the instants marked by the two hairlines were stored in files together with the SIFS onset time for further processing. The first two seconds of the fixation period were excluded as were epochs affected by blinks, eye squeezing, or technical artefacts whose durations were noted to be accounted for when calculating frequencies of SIFS occurrence and displacement rates.

### Data processing

Individual SIFSs were treated as vectors (**S**) with amplitude A and direction α relative to the right horizontal (Fig. 1F). Each consecutive pair {**S**_n_, **S**_n+1_} of SIFSs with amplitudes A_n_ and A_n+1_ and directions α_n_ and α_n+1_ was classified either as a *staircase pattern* (STC) if α_n_ and α_n+1_ differed by less than 90° clockwise or counterclockwise and otherwise as *back-and-forth pattern* (BAF). As illustrated in Fig. 1F for *n* = 1, STC patterns were characterised by the vectorial average of **S**_n_ and **S**_n+1_ and BAF patterns by that of **S**_n_ and -**S**_n+1_. Hence, if **S**_n_ and **S**_n+1_ are coplanar, patterns have an amplitude of (A_n_ + A_n+1_)/2 and are assigned the direction of the leading SIFS (α_n_). Despite this averaging, when all patterns STC and BAF are coplanar, their amplitudes sum up to the same value during the fixation period as those of individual SIFSs since each **S**_n_ occurs twice in this analysis, first in {**S**_n-1_, **S**_n_} and thereafter in {**S**_n_, **S**_n+1_} (a small difference may occur because the first and last SIFSs are considered only once).

BAF patterns were further subdivided into *paired* (PBF) und *unpaired* ones (UBF). To be classified as paired, the vectorial amplitudes A_n_ and A_n+1_ had to be 'similar', i.e. to meet the condition 0.8 < A_n_/A_n+1_ < 1.25, and its constituent SIFSs had to move in planes differing by less than 22.5°. All other BAFs were classified as unpaired. Essentially, paired BAFs are analogues of the SWJs of the literature, except that (i) no constraint is put on the interval between their two SIFSs and that (ii) their trailing SIFS combined with the next SIFS could form another PBF pattern if the similarity conditions were met (marked P' in Fig. 1C). This approach was necessitated by the baseline fluctuations that could occur under the conditions of clinical examination which made it sometimes difficult to decide whether small SIFSs initiated SWJs to one side or terminated SWJs to the other side. Identifying SWJs based on interval durations proved to be unreliable as inter-SWJ intervals could be as short as intra-SWJ intervals (cf. last P' in Fig. 1A).

In addition to the vectorial amplitude (*Amp*), three more parameters were defined to quantitatively characterise patterns as well as individual SIFSs: (i) frequency of occurrence (*Frq*), i.e. number of patterns (or SIFSs) per fixation time (30.1 s, or less if artefacts had to be excluded), (ii) displacement rate (*DisR*), i.e. cumulated vectorial amplitudes of patterns (or SIFSs) divided by the fixation time and (iii) interval (*Iva*), expressed as difference between the onset times of consecutive SIFSs. The central values of parameters *Iva* and *Frq* of individual SIFSs and those of the aggregate of all patterns (i.e. STC and BAF pooled) are identical by definition. Moreover, the more the patterns' leading and trailing SIFSs are coplanar, the better their central *Amp* and *DisR* values match those of individual SIFSs.

### Statistics

IBM SPSS 28 was used for statistical testing. In all groups, the primary SIFS parameters (*Amp*, *Iva*) turned out to have mostly non-normal distributions. Unless otherwise stated, we therefore report grand medians (sample medians of individual median values) to characterise their central tendencies, and sample medians for *Frq* and *DisR*. To test for the effect of *group*, the Kruskal–Wallis test was used followed, if significant, by Mann–Whitney-*U* tests for the post hoc search of differences between groups. To check for the effect of *pattern*, Friedman’s test was used followed by Wilcoxon tests of paired samples for the post hoc search of differences between patterns. For correlation analyses, Spearman’s rank correlation (rho) was used. Two-sided error probabilities p < 10^–2^ will be considered as significant; only three p levels (10^–2^, 10^–3^, 10^–4^) will be reported although values down to 10^–6^ were obtained. Error probabilities were corrected for multiple testing using the Bonferroni–Holm method. Error probabilities not noted in the text can be gleaned from Table 2.

**Table 2:** Sample medians of SIFS pattern amplitude, interval, frequency, and displacement rate as functions of group (CTR, ALS, PSP) and pattern (STC, BAF, UBF, PBF); patterns STC and BAF taken together represent the aggregate of all SIFS patterns; patterns UBF and PBF are subsets of BAF

	Amplitude [°]			Interval [ms]		
	CTR	ALS	PSP	CTR	ALS	PSP
***All patterns (STC and BAF)***	_***c***_***0.35***	***0.41***	_***c***_***0.91***	***464***	***409***	***367***
Staircase (STC)	0.21	_a_0.35	_c_0.85	964	798	812
Back and forth (BAF)	0.35^β^	0.47^γ^	_c_0.92	382^γ^	318^γ^	325^γ^
Unpaired BAF (UBF)	0.35	0.44	_c_0.91	410	361	341
Paired BAF (PBF)	0.40	0.54^γ^	_c_1.08^β^	298^α^	298^γ^	285^β^

	Frequency [1/s]			Displacement rate [°/s}		
	CTR	ALS	PSP	CTR	ALS	PSP
***All patterns (STC and BAF)***	_***b***_***1.24***	***1.60***	***1.82***	_***c***_***0.36***	_***a***_***0.95***	_***b***_***1.68***
Staircase (STC)	0.33	0.38	0.31	0.09	_a_0.15	0.24
Back and forth (BAF)	0.83^γ^	1.22^γ^	_a_1.61^β^	0.28 ^γ^	_a_0.75^γ^	_c_1.45 ^γ^
Unpaired BAF (UBF)	0.92	0.92	0.98	0.21	_a_0.50	_a_0.86
Paired BAF (PBF)	0.17^γ^	0.25^γ^	_c_0.52^α^	0.07^γ^	_a_0.16^γ^	_c_0.57

Rows with ***bold italic*** print show the sample medians of the aggregate of all patterns; these values are virtually identical to the sample medians of individual SIFSs. Regular print shows medians for each combination of group and pattern. Symbols a, b and c in columns ALS and PSP indicate two-sided corrected *p* < 10^–2^, < 10^–3^, and < 10^–4^, respectively, with regard to the adjacent left column (i.e. group differences ALS–CTR and PSP–ALS), those in column CTR apply to the differences PSP–CTR (only calculated for data pooled across patterns); α, β and γ, corresponding *p* values with respect to adjacent upper row (pattern differences)

We first considered the primary parameters (*Amp*, *Iva*) furnished by the interactive marking of *individual* SIFSs. As expected, the values of these parameters were virtually identical to those obtained from the aggregate of all SIFS *patterns* (i.e. STC and BAF pooled). The grand medians of *Amp* differed by 3% at most between patterns and individual SIFSs in all groups. Since the intervals of patterns are identical to those between individual SIFSs, descriptions in terms of *individual* SIFSs and of SIFS *patterns* are equivalent when only the effect of groups is of interest. Before dealing below exclusively with grand *medians* of *Amp* and *Iva* we note here their grand *averages* for comparison with other reports: *Amp*, 0.45, 0.85, and 1.33° in CTR, ALS and PSP, respectively; *Iva*, 1011, 709 and 643 ms, respectively.

## Results

### Effect of group

**Fig. 2:**
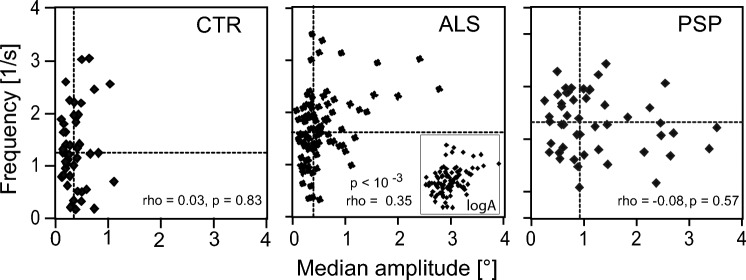
Scatter plots of relation between frequency and amplitude of individual SIFSs; each symbol represents one subject. Dashed lines indicate sample median values of frequency and amplitude, respectively. rho, Spearman's coefficient of correlation; inset logA of panel ALS uses logarithmic amplitude scale to better visualise this rank correlation

Group significantly affected *Amp*, *Frq*, and *DisR* (*p* < 10^–4^, 10^–2^, and 10^–4^, respectively). The three parameters increased in the order CTR < ALS < PSP, but not all group comparisons ALS–CTR or PSP–ALS, respectively, reached significance upon post hoc testing. In contrast, the difference between PSP and CTR was significant with all three parameters (Table 2, horizontal rows with bold italics). *Iva* exhibited no group effect, but had a weak tendency to decrease on going from CTR to ALS and PSP. The groups differed clearly regarding the joint distributions of *Amp* and *Frq* (Fig. 2). The variability of *Frq* in terms of its 90% range decreased in the order CTR > ALS > PSP (2.8, 2.2, and 1.7 s^−1^, respectively), whereas the reverse applied to the 90% range of *Amp* (0.8, 1.4, and 2.7°). Moreover, whereas *Amp* and *Frq* covaried in the ALS group (rho = 0.35, *p* < 10^–3^), they were completely uncorrelated in CTR and PSP (|rho|< 0.08, *p* = 0.57).

### Effect of pattern and group × pattern interaction

**Fig. 3:**
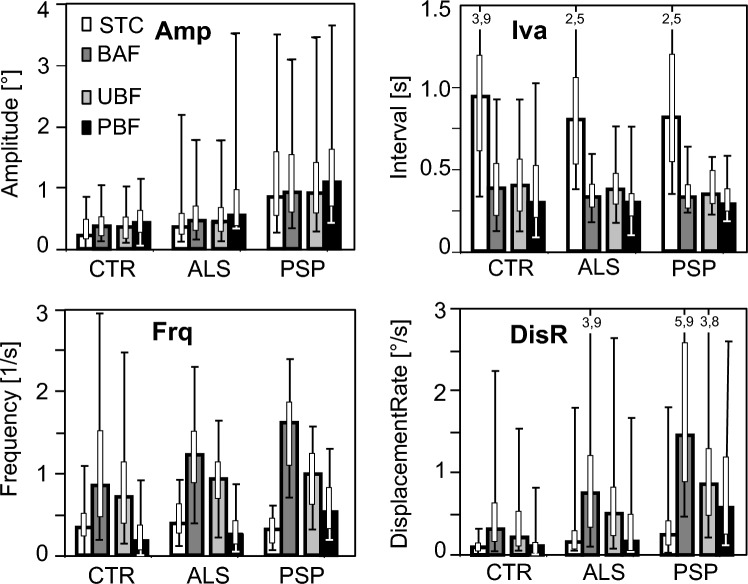
Variation of pattern parameters with type of pattern and subject group. **Amp**, grand median of individual pattern amplitudes; **Iva**, grand median of individual pattern intervals; **Frq**, median frequency of pattern occurrence; **DisR**, median displacement rate. Bold bordered bars represent grand medians or medians, respectively; whiskers show 5% and 95% quantiles (values beyond panel limits are indicated by numbers); slim bars span second and third quartiles. *STC* staircase pattern, *BAF* back and forth, *UBF* unpaired back and forth, *PBF* paired back and forth. First pair of columns compares STC with BAF; second pair compares the UBF and PBF subsets of BAF. For significances see Table 2

Across groups, pattern was a significant factor for *Amp*, *Frq*, *DisR,* and *Iva*. STC patterns had lower *Amp*, *Frq* and *DisR* and longer *Iva* than BAF patterns, and the paired subset of BAF patterns (PBF) had lower *Frq, DisR*, and *Iva* than the unpaired subset (UBF), but larger *Amp* (all *p* < 10^–4^). Many, but not all, of these pattern effects are observed also at the level of the individual groups (Table 2, significance marks α, β, or γ). Likewise, many of the group effects reported above also applied to individual patterns (Table 2 significance marks a, b, or c), but especially some differences between ALS and CTR did not reach significance. Qualitatively, the variations of *Amp*, *Iva*, *Frq*, and *DisR* are best appreciated by inspection of Fig. 3. As evident from Fig. 3 and Table 2, the amplitudes, frequencies, and displacement rates of BAF patterns reached their largest values in PSP.

A significant interaction between group and pattern occurred with *Frq* and *DisR* and concerned the fraction of the PBF and UBF patterns, respectively, among BAF patterns. For example, in both CTR and ALS, the median *Frq* of PBF patterns amounted to 20% of the BAF frequency, whereas it reached 33% in PSP (PSP *vs* ALS, *p* < 10^–4^), and a similar result held for *DisR*. Thus, the PSP group stood out also by a larger percentage of PBF patterns than seen in either CTR or ALS.

### Effects of SIFS amplitude

**Fig. 4:**
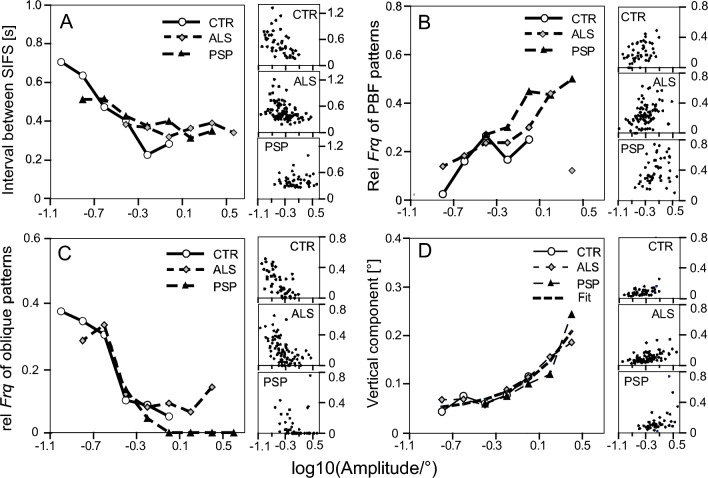
Characteristics of SIFSs in groups CTR, ALS and PSP as functions of the vectorial amplitude of SIFSs (note logarithmic amplitude scales; ticks -1.1 and 0.5 correspond to 0.08 and 3.16°, respectively). **A** Interval between SIFSs; **B** relative frequency of occurrence of PBF patterns (analogues of SWJ); **C** relative frequency of occurrence of oblique patterns; **D** unsigned amplitude of vertical component of individual SIFSs. Scattergrams to the right of each panel show the original data separately for each group (each dot represents one subject). Symbols in panels A–D represent median values of these data within amplitude bins of 0.2 logarithmic width that contain at least three values. Note the approximately common course of the curves in all groups. Thin dashed curve in D plots heuristic first-order fit to the characteristics of the three groups (vertical component = 0.044° + 0.066 • amplitude; non-linear appearance due to logarithmic abscissa)

Several parameters of both *individual* SIFSs and of SIFS *patterns* were found to depend on the patterns' amplitude in a way that was similar among all three groups. Because the range of *Amp* differed between groups, the similarity became evident only in synopses combining the data from all three groups. Figure 4 presents four examples of parameters whose variation as a function of the amplitude appeared to obey common laws in CTR, ALS, and PSP. Panel A depicts the grand medians of the interval duration in CTR, ALS, and PSP, respectively, as functions of the SIFS amplitude (values bin-wise extracted from the scattergrams flanking the panel, cf. figure legend). Considering each group separately, *Iva* decreased with increasing *Amp* in both CTR (*p* < 10^–3^) and ALS (*p* < 10^–4^), but was scattered about an approximately constant level in PSP. However, the synopsis suggests a common law such that the intervals decrease as *Amp* rises, until reaching a constant minimum of about 300–500 ms when *Amp* exceeds a threshold of the order of 0.6°. Likewise, panel B demonstrates that the percentage of paired back-and-forth patterns increases with rising *Amp* along approximately similar trajectories in all three groups. According to panel C, the percentage of oblique SIFSs (patterns occupying angular sectors centred on either 45, 135, 225, or 315°) decreases with *Amp* in all groups in a similar way except possibly for a different behaviour of ALS at amplitudes ≥ 1°. An analogous behaviour held for the contribution of oblique SIFSs to the total displacement rate of SIFSs (not illustrated). Finally, the magnitude of the vertical component of single SIFSs depended on *Amp* in an almost identical manner in all groups (panel D), exhibiting little variation at *Amp* values < 0.6° and a gradual increase beyond this threshold.

### Pattern orientation

In all groups, patterns falling into oblique or vertical sectors had considerably reduced amplitudes and frequencies as compared to those within the two horizontal sectors. Accordingly, their displacement rates made only small or negligible contributions to the total *DisR* (oblique patterns, 12, 16 and 8% in CTR, ALS, and PSP, respectively; vertical, 3, 4, and 1%). The direction orientation of patterns falling into the left or right horizontal sectors deviated slightly from an exactly horizontal alignment. In all groups, most rightward patterns had a small downward orientation α_R_, whereas leftward patterns had an upward one (α_L_), suggesting a common, slightly inclined plane. Inclinations, calculated from (α_R_ + α_L_-180)/2, ranged from virtually zero up to 2° clockwise in our three groups. None of these inclinations were significantly different from zero nor did they differentiate between groups or patterns.

## Discussion

Before considering how and why the amplitude of SIFS determines the probability of SWJ coupling, we first briefly compare our primary data (*Amp* and *Frq* of *single* SIFSs) to previously published results and comment on the variations of the parameters *Amp*, *Iva*, *Frq*, and *DisR* across groups and patterns. Finally, we will touch on the question of where in the brain the alterations of SIFSs in ALS and PSP could arise.

### Amplitude and frequency of individual SIFSs

Although our way of measuring the amplitude of SIFSs ignored the dynamic overshoot of SIFSs and considered only the final eye displacement, the values of *Amp* obtained in CTR, ALS, and PSP compare well with recent studies that mostly seem to have included dynamic overshoots. Our average *Amp* of 0.45° in CTR is in good agreement with the 0.3° to 0.6° range reported by others (e.g. Donaghy et al. 2009; McGivern and Gibson 2006; Nij Bijvank et al. 2019; Otero-Millan et al. 2011; Pinnock et al. 2010). For ALS, we obtained a geometric average of 0.61° in close agreement with the 0.64° of Donaghy (2009), and the average *Amp* of PSP patients (1.33°) is within the reported range of about 0.8°–1.5° (Garbutt et al. 2004; Otero-Millan et al. 2013; Pinnock et al. 2010).

The observation that the 90% ranges of the subjects' SIFS amplitudes and SIFS frequencies, respectively, exhibited inverse differences between CTR and PSP (*Amp* range larger in PSP, *Frq* range larger in CTR) had not been anticipated. The fact that the dispersion of *Frq* was conspicuously smaller in PSP than in CTR despite a significantly larger median value is of note. In a complementary study we found *Frq* to be uncorrelated with the extent of the PSP patients' eye movement palsy (Becker, in preparation). Thus, in comparison to controls, the SIFS *frequency* of PSP patients appears to be 'switched' to a higher, but in all patients relatively stereotyped level which, in terms of vertical oculomotor performance, does not depend on the severity of the disease. In contrast, their SIFS *amplitudes* were found to increase with severity as testified by a significant negative covariation with vertical eye movement palsy. These differences explain the lack of covariation between *Amp* and *Frq* in PSP and hint at the possibility that the alterations of *Amp* and *Frq* arise from separate structures in PSP.

### Patterns

*Comparison between groups*. The amplitude and the frequency of the aggregate of SIFS patterns (i.e. STC and BAF) and by the same token those of individual SIFSs were slightly larger and more frequent in ALS than in CTR and considerably larger in PSP (cf. Table 2 and Fig. 3). These results agree with previous work. All studies of PSP, whether focused on SWJs only (Garbutt 4212; Rascol 3430; Troost 2440) or considering all types of SIFSs (Otero-Millan et al. 2011), report a considerable increase of *Amp* and *Frq* in comparison to controls, whereas clearly smaller increases of *Amp* have been found in ALS (Donaghy et al. 2009; Shaunak et al. 1995). In a preliminary study comparing ALS patients to controls (Becker et al. 2019), we had also observed a small but significant increase of the amplitude, in contrast to the present study where the increase was not significant. Moreover, based on the uncorrected p-value, the preliminary study found no increase of SIFS frequency in ALS; however, with the present data, the uncorrected *p,* unlike the corrected one, would suggest a significant increase (p_uncorrected_ < 10^–2^). These discrepancies have, for one thing, methodological reasons (subject groups not identical, preliminary data based solely on horizontal BAF patterns) and also reflect the relative smallness of the increase of SIFS activity in ALS. As a measure of the combined effect of frequency and amplitude, we have calculated the displacement rate *DisR* which, in *individual* subjects, represents the product *Frq*•*Amp* (for the *sample* medians of *DisR* this applies only approximately since multiplications and medians are non-linear operations, cf. Table 2). *DisR* provides a useful summary of disease-related changes in SIFS activity. In terms of error probability, it provided the best discrimination between groups in that the differences between any two groups were significant, whereas *Amp* reached significance in only two comparisons and *Frq* in only one (cf. Table 2). However, comparing groups only in terms of *DisR* may conceal differences concerning the interrelation between *Amp* and *Frq*.

Unlike amplitude and frequency and in agreement with Donaghy (2009) and Pinnock (2010), the interval durations did not significantly differ between groups and thus failed to reflect the increase of *Frq* in the patient groups. The reason is twofold: (i) the median *Iva* depends not only on the number of SIFSs during a given period, but also on their temporal clustering; (ii) because of the reciprocal, and hence non-linear relationship between *Iva* and *Frq*, their sample averages and medians are not reciprocals of each other. Thus, the intervals cannot be used as surrogates for the frequency of SIFS occurrence.

*Characteristics of patterns*. Staircase patterns arise in two situations (cf. Fig. 1B and C): (i) the trailing saccade of a BAF pattern into one direction is followed by the leading saccade of a BAF or STC pattern in opposite direction; (ii) a mostly small ipsidirectional saccade follows upon a larger one as if a correction was intended. The lower frequency of STCs in comparison to BAFs indicates that SIFSs frequently occurred as trains of deviations to, and back from, always the same side of the fixation point with only occasional changes of side (scenario i). However, the smaller amplitude of STCs as compared to BAFs suggests that not all STC patterns resulted from such a change of side, but were also due to small corrections (scenario ii). Multistep staircases as reported in some patients with IPD (Shaikh et al. 2011) occurred in none of our groups.

As explained in Methods, paired BAF patterns (PBFs) are qualitative equivalents of SWJs, but differ from SWJs regarding two aspects: (i) also the SIFSs framing inter-SWJ intervals form PBF patterns if they meet the PBF similarity criteria; (ii) PBF patterns are not subject to constraints regarding interval duration. Yet, despite the inclusion of inter-SWJ intervals, the PBF sample *medians* of *Iva* (range 285–295 ms, cf. Table 2) were almost identical to reported averages of *intra*-SWJ intervals in CTR and PSP (e.g. 280 – 290 ms, Otero-Millan et al. 2011). However, the inclusion of *inter*-SWJ intervals became noticeable in the form of longer sample *averages*. (CTR, 466; ALS, 318; PSP, 323 ms). Because of the inclusion of inter-SWJ intervals meeting the PBF criteria, our PBF frequency figures are likely to be larger than those of SWJ if these were identified using the present amplitude and direction criteria. However, despite the inclusion of inter-SWJ intervals, the PBF frequency figures obtained in CTR (0.17 s^−1^) and PSP (0.56 s^−1^) were not larger but slightly lower than the reported averages of SWJs in CTR (0.2–0.44 s^−1^, Garbutt et al. 2004; Nij Bijvank et al. 2019; Otero-Millan et al. 2011) and PSP (0.8 s 1, Otero-Millan et al. 2011). This discrepancy is not caused by our comparing median values to averages since it emerges also if the *relative* frequencies of PBF patterns (= *Frq*_PBF_ / Frq_all_patterns_) of CTR and PSP (0.14 and 0.29, respectively) are compared to those of SWJs (0.18 and 0.38 s^−1^, calculated from data of Otero-Millan 2011). Thus, our straightforward PBF criteria (amplitude difference between successive SIFSs < 20%, direction difference < 22.5°) imposed tighter constraints than those used in SWJ studies but could easily be modified if a larger percentage of SIFS patterns were to be defined as PBF.

### Dependence of SIFS characteristics on amplitude: the role of noise

In healthy subjects and patients affected by different Parkinsonian syndromes or by spinocerebellar ataxia, the probability of SWJ occurrence has been found to depend on the amplitude of the subjects' SIFSs (Otero-Millan et al. 2013). The present analysis extends and complements this observation by showing that (i) also in ALS the relative frequency of occurrence of our SWJ surrogates, i.e. of PBFs, varies with SIFS amplitude and that (ii) in all groups examined here the amplitude determines also the spatial orientation of SIFSs, the magnitude of their vertical component, and the interval duration. Moreover, when viewed together, the relationships of these parameters to *Amp* in CTR, ALS, and PSP, respectively, turn out to be pieces of general laws that appear hold for an *Amp* range of 0.1° to a least 3°, irrespective of group. We suggest that these laws can be explained by the presence of a constant background component of physiological and technical noise that does not vary with *Amp* (henceforth referred to as 'constant component'). By 'noise', we here understand all factors causing random deviations of the recorded amplitude and direction from their presumably intended magnitude. Physiological noise mainly arises from four sources: (i) the intrinsic variability of the mechanism generating SIFSs, (ii) drifting movements of the eyes between SIFSs which modify the SIFS amplitude and the direction required to reach a desired gaze position, (iii) insufficient head stabilisation evoking compensatory eye movements, and (iv) a partial occlusion of the pupil by upper eyelid drooping in some subjects which degrades the quality of vertical eye position recording by video-oculography. The technical noise of various VOG instruments (but not of the presently used EyeSeeCam) has been examined by Holmqvist and Blignaut (2020) who recorded with these devices accurately calibrated movements of artificial eyes. They found their gain to wax and wane as the dummy eyes are rotated laterally thus causing wrong position measurements. These inaccuracies are irrelevant in the present context since the leading and trailing SIFSs of a correctly commanded PBF pattern would undergo identical distortions. Of more importance is the smallest detectable displacement (i.e. the resolution) because it scales with the magnitude of the spontaneous fluctuations of the VOG signal that cause the random distortions of SIFS measurements.

Finally, our way of determining the amplitude of SIFSs causes a particular form of vertical noise in subjects with vertical drifts of the recorded eye position. Since we refer the amplitude of SIFSs to the steady state reached *after* the period of postsaccadic dynamic processes, the vertical displacement from drifts during this period is included in the measurement of the vertical SIFS component.

Our arguments are best illustrated by proceeding from the assumption that under the conditions of the present study (fixation at a single spot on a structureless background), the initial deviations of SIFSs from the fixation base line are ‘intended’ to be horizontal, in agreement with the horizontal dominance of SIFSs found here and in most previous studies. Clearly, the smaller the desired magnitude of a horizontal SIFSs is in comparison to a concomitant vertical noise component, the more its direction will be modified in an unpredictable way. For example, if the vertical component happens to be of same magnitude as the horizontal one, an oblique saccade with a direction of 45° will result. The dispersion of the landing positions of saccades towards targets of 0.1° eccentricity observed by Poletti (2020) suggest that the intrinsic physiologic variability alone can turn many intended horizontal saccades of 0.1° into an oblique sector. However, the larger the intended horizontal SIFS is, the less vertical noise can modify its direction unless it does not rise at the same rate as the SIFS amplitude. This was not the case, however. The effective vertical noise is identical to the unsigned vertical component of SIFSs. As shown in Fig. 4D, this component remained essentially constant up to *Amp* magnitudes of about 0.6°, in good agreement with the dispersion of the endpoints of small targeted saccades (Poletti et al. 2020)). According to a heuristic first-order fit to the experimental data, the grand median of the vertical noise can be approximated by 0.044° + 0.066 • *Amp* (Fig. 4D, heavy dashed curve), which means that (i) its rate of increase is far below unity and that (ii) its constant component (0.044°) dominates up to about 0.67° (= 0.044°/0.066). According to this approximation, half of the SIFSs with an intended *Amp* of 0.1° would deviate by more than 20° up or down from horizontal. Thus, a considerable percentage of deviations can be expected to exceed 22.5° and turn the SIFSs into an oblique direction sector. In contrast, the median deviation of large SIFSs with an *Amp* of, say, 4° would amount to only about 4.5°, which reduces the chance that individual cases reach more than 22.5°. The vertical component of SIFSs has also been examined by Otero-Millan et al. (2011). Regarding PSP, a rough estimation of their data suggests a similar variation with *Amp* (0.063° + 0.092 • *Amp*) as observed here, whereas the apparent lack of a constant component in their controls is clearly at variance with the present data.

From the above, we conclude that the strong influence of the constant component of vertical noise upon the direction of the smallest SIFSs reduces the probability that successive low amplitude SIFSs meet the PBF (or SWJ) criteria of similar motion planes, hence the low rate of these patterns when *Amp* is small and its increase when *Amp* becomes larger (Fig. 4B) However, not only vertical noise, but also noise along the intended horizontal direction is a factor in shaping the relationship between PBF frequency and SIFS amplitude. Unlike the effective vertical noise, no direct estimate of the effective magnitude of horizontal noise is available. Yet, it is beyond doubt that horizontal noise also comprises a constant component. Regarding the physiological contribution to horizontal noise, this contention is supported by the precision of small targeting saccades which is approximately constant for intended amplitudes of 0.1–0.3° (Poletti et al. 2020). Moreover, the contribution of instrumentation noise inevitably has a constant background component. Therefore, by similar arguments as above, as long as the intended amplitudes of consecutive SIFSs are as small as the effective horizontal noise, there is also little chance to meet the amplitude criterion for PBF (or SWJ).

In summary, we suggest that the different prevalence of PBF or SWJ patterns among groups results from the interaction of the group-specific magnitudes of *Amp* with constant physiological and technical noise components whose *relative* impact on SIFS amplitude and direction is large in the small amplitude range (as in control subjects), but decreases with larger amplitudes (as in PSP). Remarkably, the noise appeared to be similar in all three groups; otherwise, their curves in Fig. 4B–D would be vertically displaced from each other rather than overlap. We have not attempted to separate the contributions of physiological and technical noise, respectively. As noted above, the vertical SIFS component presumably represents the effective overall vertical noise. Its particularly impressive similarity across groups (Fig. 4D) might reflect a dominant role of technical noise, which, obviously, does not depend on subject groups, but is determined by the experimental setup. The fact that VOG recordings of vertical eye movements are noisier than those of horizontal ones (Choe et al. 2016; cf. also Fig. 1A–C) may have contributed to overshadow physiological group-specific difference of vertical noise. However, in view of the similarity of the presently obtained vertical SIFS component to that observed by Otero-Millan et al. (2011) in PSP patients, we presume that our measurements of this component were at least not heavily affected by the inclusion of the vertical drift occurring during the period of postsaccadic dynamic processes (cf. above).

Constant noise components of physiological and technical origin will occur in any recording of SIFSs. Hence, whichever healthy or diseased subjects are considered, their percentage of SWJs will increase with the amplitude of their SIFSs unless there are severe deficits of the visual fixation system.

Unlike the orientation and the occurrence of PBFs and SWJs, the variation of the interval duration as a function of SIFS amplitude is not an effect of oculomotor and technical noise. The decrease of *Iva* with increasing *Amp* (Fig. 4A) is reminiscent of the latency of refixation saccades which decreases from a central peak at small target eccentricities to a constant minimum at intermediate eccentricities (Kalesnykas and Hallett 1994). This similarity can be expected, since centripetal SIFSs are likely to be refixation saccades elicited by the apparent target displacement from the preceding centrifugal SIFSs which scales directly with their amplitude. However, since our measure of *Iva* includes also the intervals *preceding* spontaneous centrifugal SIFSs, the *Iva* versus *Amp* characteristic (Fig. 1A) is displaced by an estimated 100–150 ms towards larger values with respect to that of purely reflexive saccades.

### Preferred motion plane of SIFSs

The percentage of SIFSs falling into one of the two horizontal direction sectors grew as the amplitude of SIFSs increased. Within these sectors, the average motion planes were clockwise rotated by less than 2° from horizontal in all groups. Therefore, we conclude that human SIFSs occurring under the present conditions are ‘intended’ to be horizontal. A slight clockwise rotation of the SIFS plane has also been observed in healthy subjects of similar age as ours (Otero-Millan et al. 2011). However, in rhesus monkeys SIFSs occur predominantly in the vertical plane (Costela et al. 2015; Cui et al. 2009), although their oculomotor system closely resembles the human one. This difference suggests a functional role of SIFSs related to different, yet to be elucidated, behavioural demands in the two species. Therefore, it would be helpful to find in healthy subjects a correlation between the characteristics of SIFSs and indices of visuo-spatial and attentive performance at the *interindividual* level.

### Origin of SIFS alterations

There exists no secure knowledge regarding the site(s) and the mechanism(s) of SIFS generation in healthy subjects. One theory proposes that SIFSs are triggered by random fluctuations of the balance between the activities of the left and right rostral fixation zones of the bilateral superior colliculi (SC) which would simulate the existence of targets of interest close to the fovea (Hafed et al. 2009; Hafed 2011; Otero-Millan et al. 2018).

In PSP, basal ganglia (including putamen, subthalamic nucleus, and substantia nigra) and midbrain areas are strongly affected (Kovacs et al. 2020). Therefore, based on the above theory, it has been proposed, that (i) the elevated frequency in PSP results from a loss of inhibitory SC control by the basal ganglionic substantia nigra pars reticulata (SNpr) and (ii) the enlarged amplitudes result from a partial disinhibition of omnipause neurones due to a demise of inhibitory projections from the vertical burst neurones (vBN) of the midbrain (for details see Otero-Millan et al. 2013). The lack of correlation between *Amp* and *Frq* in PSP is compatible with the hypothesis that *Amp* and *Frq* are being modified at different sites. Moreover, as mentioned above, *Amp* correlates with the severity of vertical eye movement palsy in keeping with the suggestion that its increase is secondary to the loss of vBN. However, it is difficult to see why the fluctuations of SC activity after loss of inhibition by SNpr should occur exactly along the collicular representation of the horizontal plane. According to the results of pharmacological inactivation of SNpr in the monkey (Hikosaka and Wurtz 1985), one would expect random fluctuations across the 2-D collicular space and, hence, a large portion of predominantly vertical SIFSs instead of the observed small percentage. Thus, the putative loss of inhibition by SNpr may only concern the temporal aspect of SIFS activity, whereas their horizontal orientation would still be ensured by some form of higher control.

Regarding ALS, the small increase of the amplitude beyond the level of controls cannot be attributed to a loss of vBN. The vertical refixation saccades of our ALS patients were as fast as those of CTR, indicating a normal state of their vBN. At present, the most likely speculation explains the alteration of *Amp* and *Frq* in ALS by the degeneration of frontal cortical areas. For example, reversible cooling experiments (Peel et al. 2016) suggest that an impairment of the frontal eye fields may result in enlarged SIFS amplitudes. The observed covariation of *Amp* and *Frq* in ALS (which should be confirmed by a control study) is compatible with an origin of their alterations in neighbouring cortical areas. A candidate structure is the dorsolateral prefrontal cortex (DLPFC) which assumes a role in inhibiting inappropriate behaviours (Pierrot-Deseilligny et al. 1991; Ploner et al. 2005). The affection of the prefrontal cortex by ALS pathology (Braak et al. 2013) might cause a disinhibition of saccade triggering structures, thus raising the frequency of SIFSs (Shaunak et al. 1995). Finally, despite the profound differences between ALS and PSP, the pathologies of both disorders invade frontal areas and cause frontal dysfunctions. Hence, despite the lack of covariation between *Amp* and *Frq* in PSP, the possibility that the alterations of *both* parameters have a frontal cause in PSP as well cannot be excluded as an alternative to the basal ganglionic theory mentioned above.

## Conclusion

In humans, the small involuntary fixation saccades (SIFSs) occurring during fixation at a small target on a uniform structureless background are 'intended' to be horizontal when leaving the required line of sight and to be offset by successive mirror-reversed SIFSs which, ideally, would always return gaze to the required line of sight, thus creating square wave jerk (SWJ) patterns. However, in the low amplitude range of SIFSs, this intention is foiled by a constant, amplitude-independent component of physiological and technical noise of similar magnitude as the intended SIFSs. With larger SIFS amplitudes, the impact of this noise component decreases so that the chance to approximately meet the 'mirror criterion' rises. The resulting dependence of the probability of SWJ occurrence on SIFS amplitude is not only qualitatively similar in healthy controls (CTR) and the two clinically unrelated diseases ALS and PSP, but appears to also quantitatively follow a common law. According to this law, the probability of SWJ occurrence depends only on the SIFSs amplitudes individuals produce but not on which group (CTR, ALS, PSP) they belong to. As a corollary, we conclude that the constant noise component was similar in all three groups. As the noise of SIFS recordings will always comprise an amplitude-independent component, SJW probability will rise with SIFS amplitude in any group of subjects, whether healthy or diseased (except for cases of severe dysfunctions of the visual fixation system). Finally, the 2-D distributions of SIFS frequency versus SIFS amplitude appear to be different in each group; a covariation between amplitude and frequency occurred only in ALS. The lack of a covariation in PSP might indicate that the disease related increases of SIFS amplitude and frequency, respectively, occur at different sites in this disorder.

## Data Availability

Upon request, the data underlying the reported results are available from the corresponding author.

## (Italic print marks result parameters)

ALS: Amyotrophic lateral sclerosis
*Amp*: Vectorial amplitude
BAF: Back-and-forth pattern
CTR: Control subjects without known neurological impairments
*DisR*: Displacement rate
*Frq*: Frequency of occurrence
*Iva*: Interval
PBF: Paired back-and-forth pattern
PSP: Progressive supranuclear palsy
**S**_i_: Vector representing the ith SIFS
STC: Staircase pattern
UBF: Unpaired back-and-forth pattern
